# Fault Diagnosis of Rolling Bearing Based on Fast Nonlocal Means and Envelop Spectrum

**DOI:** 10.3390/s150101182

**Published:** 2015-01-09

**Authors:** Yong Lv, Qinglin Zhu, Rui Yuan

**Affiliations:** School of Mechanical Engineering, Wuhan University of Science and Technology, Wuhan 430081, China; E-Mails: lvyong@wust.edu.cn (Y.L.); 201403703005@wust.edu.cn (R.Y.)

**Keywords:** fast NL-Means, envelop spectrum, fault diagnosis, rolling bearing

## Abstract

The nonlocal means (NL-Means) method that has been widely used in the field of image processing in recent years effectively overcomes the limitations of the neighborhood filter and eliminates the artifact and edge problems caused by the traditional image denoising methods. Although NL-Means is very popular in the field of 2D image signal processing, it has not received enough attention in the field of 1D signal processing. This paper proposes a novel approach that diagnoses the fault of a rolling bearing based on fast NL-Means and the envelop spectrum. The parameters of the rolling bearing signals are optimized in the proposed method, which is the key contribution of this paper. This approach is applied to the fault diagnosis of rolling bearing, and the results have shown the efficiency at detecting roller bearing failures.

## 1. Introduction

Nowadays, the rolling bearing has been widely used in the applications of modern industrial society, and its working conditions are of vital importance. Rolling bearing faults appear with a great incidence, due to its complexity and poor working conditions, and bearing signals are usually drowned by noises in practice, which make fault diagnosis difficult [1,2]. Therefore, it becomes an important point to reduce the interferences of noise more effectively, which has attracted great attention from a great many scholars.

Recently, many methods have been proposed for signal denoising, such as wavelet threshold [3], blind source separation [4] and singular value decomposition [5]. These methods make great contributions to fault diagnosis. They all adopt the procedure of decomposition and reconstruction [6] and can effectively separate the noises and the original signals. However, the decomposition methods that extract the main information of the original vibration signals would cause signal distortion of the local information and loss of details. Additionally, they need the original data to meet certain mathematical laws. The actual signals often show strong randomness, so the decomposition and reconstruction would cause inappropriate reduction.

The denoising problem is a critical issue in image processing. The transmission and recording will be affected by a variety of interferences in the process [7], such as Gaussian noise [8] and impulse noise [9]. In traditional image denoising technologies, the methods are only for local areas and would cause large deviations for local statistical texture areas of rich information. These methods effectively work on removing noises in homogeneous areas, but cannot retain complete image structure information, while always making the edge details fuzzy. Subsequently, Donoho [10], Minh Do [11], Chen, S. [12], *etc.*, put forward the image denoising methods based on wavelet, conturlet and SureShrink. They are widely used due to their advantages of multi-resolution and multi-scales, but the essence that a fixed window works in local areas has already been used in traditional methods; meanwhile, they cause the production of false information under the influence of basis functions. At the same time, they will produce the Gibbs' phenomenon on the image edges [10–12]. A recent approach regarding image denoising that aims at solving the edge problem is the nonlocal means filter (NL-Means), introduced by Buades *et al.* in 2005 [13].

This method overcomes the limitation of the local neighborhood filter by using the characteristics of the similar image blocks to restore the original image without the regularity premise, which is a necessary criterion of traditional image denoising. The weights do not depend on the distance between two points, but the Euclidean distance between the neighborhood levels [13–15]. They effectively eliminate the artifact and edge problems, meanwhile retaining the most image details. The NL-Means denoising results are not the presentation of mathematical functions, but the good reduction of original images, so this method consequently causes a small degree of distortion.

The basic idea of NL-Means is that natural images generally have a large amount of information and contain some similar compositions, so we can take the weighted average to eliminate noises by finding similar patches in the search field. Through the analysis of the 1D mechanical failure vibration signals, we find that repeated patches appear in the vibration signals, and the noises are superimposed over these fault pulses randomly. The NL-Means can be used to restore the original signals instead of using the methods of decomposition to extract the fault pulse function. In [16], it can be found that NL-Means achieves a lower rate of distortion when compared to the EMD (Empirical mode decomposition)/wavelet in an ECG signal application. In this paper, we propose a patch-based method named NL-Means for rolling bearing fault diagnosis, while applying envelop spectrum analysis [17] as post-processing. The simulative and experimental results prove that this method can effectively restrain the interference of noises and accurately extract the fault characteristic frequency information of the defect bearing.

This paper is organized as follows: Section 2 presents the necessary instructions of the NL-Means algorithm and the fast NL-Means algorithm. Section 3 introduces the parameters adaptively selected in the process of the fast NL-Means algorithm by using the simulation bearing fault signals. Section 4 introduces the envelop spectrum analysis with the Hilbert transform, and specific steps of the approach are presented. Sections 5 applies fast NL-Means and envelop spectrum analysis to the diagnosis of the actual rolling bearing fault signals, and the conclusion is given in Section 6.

## 2. NL-Means Algorithm

The basic idea of NL-Means is that natural images generally have a wealth of information and contain some similar compositions [10]. The additional noises of similar patches are random. Thus, it is feasible to take the weighted average to eliminate noises by finding similar patches in the search field. Therefore, Buades *et al.* proposed the NL-Means image denoising algorithm by weighting the similarity of image neighborhoods. It is based on the fact that natural images often have duplicate modules. There are some modules in mechanical vibration signals that are repeated, and additional noises of similar patches are random, which all prove the test of the NL-Means.

### 2.1. Basic NL-Means Algorithm for 1D

The NL-Means algorithm of 1D signals [13] is introduced in this paper. NL-Means algorithm denoising aims at solving the problem of restoring the original signals in a noisy environment.

Under the additive noise models, noise signals can be expressed as follows:

(1) y(i)=x(i)+n

when *y* is the observed signals, *x* is the original signals that have not been polluted; *n* is additive white Gaussian noise.

In the NL-Means filter, *x̃*, which represents the estimate of signals *x*, is the weighted average of all of the values in the search areas. Therefore, the NL-Means filter can be defined as follows:

(2) $$\tilde{x}\left(s\right)=\frac{1}{Z\left(t\right)}\sum_{t\in M\left(s\right)}\omega \left(s,t\right)y\left(t\right)$$

when *Z*(*t*) is the normalized constant, *Z*(*t*) = Σ*_t_*_∈_*_M_*_(_*_s_*_)_ ω(*s*,*t*) correspond to a set of neighboring sites of *s*. The weight ω(*s*,*t*) represents the degree of similarity between two patches. It is given by:

(3) $$\omega \left(s,t\right)=\exp(-\frac{\sum_{\delta \in \Delta }{\left(y\left(s+\delta \right)-y\left(t+\delta \right)\right)}^{2}}{2L_{\Delta }\lambda^{2}})\equiv \exp(\frac{-d^{2}\left(s,t\right)}{2L_{\Delta }\lambda^{2}})$$

when λ is a bandwidth parameter, Δ is the patch surrounding *s* and *L*_Δ_ is the patch surrounding *t*. We define *d*^2^ (*s*,*t*) as the square of the point-by-point difference between samples in the patches centered on *s* and *t*.

### 2.2. Fast NL-Means Algorithm

Let search domain *M*(*s*) = [−*K*,*K*] and the patch *L*_Δ_ = [−*P*,*P*]. To get a more robust average, the search domain *M(s)* is defined as the entirety of the signals in the case of ignoring the complexity of the calculation. If the search domain is ideally taken to be the entirety of signals, it is a complete nonlocal average process, but in practice, the calculation method is improved to reduce the computing time. Fast NL-Means methods [10,18] are used to reduce the computing time. This approach significantly speeds up NL-means by reordering operations to eliminate a nested loop [18].

The key to saving time is to find a faster way to calculate the weight ω(*s*,*t*), and the key to calculating the weight ω(*s*,*t*) is to calculate the square of the point-by-point differences between samples in the patches centered on *s* and *t*. The signal length is *N.*

*S_dx_* is defined as the discrete integration of the squared difference of the original signal *x* and its translation by *d_x_*.

(4) $$S_{d_{x}}\left(p\right)=\sum_{k=0}^{p}{\left(y\left(k\right)-y\left(k+d_{x}\right)\right)}^{2},p\in N$$

From Equation (4), this algorithm leads to boundary effect. There are many methods to eliminate the boundary effect; in order to simplify the process without affecting denoising edges, the starting point is settled as *P* + 1 and the end point as *N* − *P*, and both sides do not participate in the calculation.

In Equation (3)*d*^2^(*s*,*t*) = Σ_δ∈Δ_ (*y*(*s* + δ) −*y*(*t* + δ))^2^. Let *d_x_* = *t* − *s*, and define *q* = *s* + δ. The patch sizeΔ = [−*P*,*P*]. Through these settings, *d*^2^(*s*,*t*) is calculated as:

(5) $$d^{2}\left(s,t\right)=\sum_{s-P}^{s+p}{\left(y\left(q\right)-y\left(q+d_{x}\right)\right)}^{2}$$

Meanwhile, combined with Equation 4,*d*^2^(*s*,*t*) is defined as follows:

(6) $$d^{2}\left(s,t\right)=S_{d_{x}}\left(s+P\right)-S_{d_{x}}\left(s-P\right)$$

The *S_dx_* is known for a given patch centered on *t*. We can calculate the weight for a fixed length of patch. The size of the patch *2P* + 1 is independent in Equation (6).

The fast NL-Means is summarized in Table 1.

## 3. Simulation of Denoising and Parameter Setting

The purpose of the NL-means is to restore the original image patch centered on a specified point in dealing with the 2D images [19,20]. The given search window is used to search for similar image patches in the whole image [21]. Their similar degrees, called the Euclidean distance, are measured by the weights [10].

**Figure 1.**
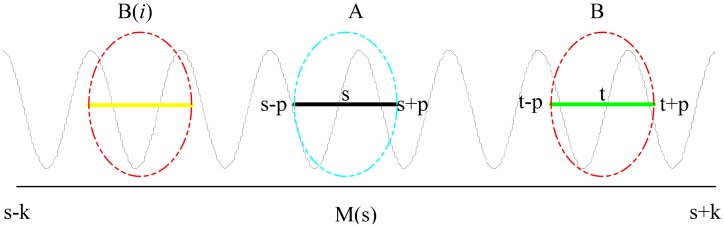
Illustration of NL-means parameters.

Parameters are examined for rolling bearing signal denoising from NL-means in the 2D image processing methods. Assume that the patch, A, is the goal to restore, which focuses on *s* as the center. The three key parameters of fast NL-Means are the width of the patch size *P* (*L*_Δ_ = [−*P*,*P*]), the size of the search domain *K* (*M*(*s*) = [−*K*, *K*]) and the bandwidth λ. The similar patch, B, has the same length as the goal patch, A. Similar degrees are measured by ω (*s*,*t*). All of the similar patches, B, are obtained from the estimated value of A. Figure 1 shows the nonlocal average algorithm parameters.

The size of patch *P* determines the size of the similar patches and the search window, so the choice of *P* should be adapted to the size of the target patch [13]. If the value of *P* is too large, it leads to the reduction of the patches with a good similar structure. The reduction of the redundant information leads the average being inadequate. If the value of *P* is too small, a large number of patches emerge, and these patches are not typical; so, they cannot show the repeat feature information of signals, which results in a more complicated calculation. A proper selection of *P* relates to whether the signals can be properly restored. For fault rolling bearing signals, the selection of *P* makes the patches contain a complete pulse waveform at least. Indeed, it is well known that the edges are not satisfactorily denoised by using NL-Means, and the size of *P* results in this problem. In this paper, a certain length of the signal on the edge is added by using the method of mirroring to expand the boundary.

The size of the search domain is dependent on *K*. In theory, for the search domain size *M*(*s*) = [−*K*, *K*], bigger is better, but the increasing of *K* will increase the calculation time. When the signals length is small (*N* < 4000), the *K* value is equal to the largest (*K* = *0.5 N*), so the search domain covers the whole signal range, in order to get the most similar patches for weighted averaging. When the signals length is large (*N* > 4000), *K* > 0.25 *N* and < 0.33 *N*, so the search domain can contain most of the signals' information, causing the number of patches in the weighted averaging and the computing time to be reduced at the same time.

In the NL-Means algorithm, the parameter bandwidth λ has a smoothing effect and plays the same role as the bandwidth for kernel methods in statistics. It determines the attenuation velocity of the exponential function. The value of λ directly decides the standing or falling of the filtering results. When λ is too small, it causes noise fluctuation, thus interferences in different similar weights lead to inadequate results, on average. When λ is too large, it causes the signal too smooth, resulting in missing signal detail [13].

**Figure 2.**
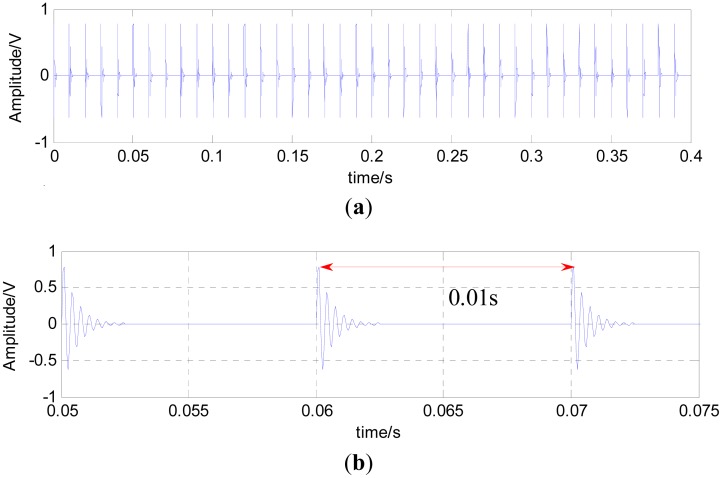
(**a**) Simulated fault signal of the rolling bearing; (**b**) local amplification of the simulated fault signal of the rolling bearing.

In order to determine these parameters more directly, fast NL-Means is used to simulate rolling bearing signals for bearing signal denoising. The simulation of the signal model of rolling bearing without race fault can be expressed as follows [22]:

(7) $$y\left(t\right)=Ae^{-\xi 2\pi f_{n}t}\sin2\pi f_{n}\sqrt{1-\xi^{2}t}$$

where *f_n_* is the inherent frequency of rolling bearing, ξ is the damping coefficient and *A* is the rolling bearing displacement constant. Here, let *f_n_* = 3000 Hz, ξ =0.1 and *A* = 1. The fault cycle is 0.01 s. The sampling frequency is 20 kHz. Figure 2a shows the simulated fault signals of the rolling bearing, and (b) is the local amplification of the simulated fault signals of the rolling bearing. The pulse signal presents a complete regularity under the condition of no noise interference.

For such impact signals, *P* = 100, so *L*_Δ_ = 200. A similar patch exactly contains one pulse cycle (0.01 s). For the simulation signals, the computing time can be appropriately increased to achieve the best results. Therefore, *K* = 4,000, but the trouble is choosing an appropriate value of λ.

Because of the difficulty in choosing this parameter, a number of scholars have done a lot of research to determine it. Ville and Kocher use the SURE-based method [23,24] to choose this parameter and find that the optimal parameter selection is λ = 0.5 σ (σ is the standard deviation of the noise. Obviously, the bandwidth λ is decided by the value of σ.). The noise standard deviation σ can be calculated by Equation (8) in the simulation. Here, for our test, we make the parameter λ = 0.6σ (an explanation is given for the choice in the following paragraphs):

(8) $$\sigma =\sqrt{\frac{\overset{n}{\underset{i=1}{\text{∑}}}{\left(x_{i}-\bar{x}\right)}^{2}}{\left(n-1\right)\log10\left(SNR\right)}}$$

where *SNR* is the value of signal-to-noise ratio, *x̄* is the mean of the given time series, *n* is the length of the time series. We also tried different values, when λ = *c*σ, *c* ∈ (0,1). In order to determine the noise reduction effect, when constant coefficient *c* transform in its domain, the improved signal-to-noise ratio index (*impSNR*) [13], mean square error (MSE), and percent distortion (PRD%) are given by:

(9) $$impSNR=10\times \log\frac{\sum_{n=0}^{N-1}{\left(y\left[n\right]-x\left[n\right]\right)}^{2}}{\sum_{n=0}^{N-1}{\left(\hat{x}\left[n\right]-x\left[n\right]\right)}^{2}}$$

(10) $$MSE=\frac{1}{N}\sum_{n=0}^{N-1}{\left(\hat{x}\left[n\right]-x\left[n\right]\right)}^{2}$$

(11) $$PRD\%-100\sqrt{\frac{\sum_{n=0}^{N-1}{\left(\hat{x}\left[n\right]-x\left[n\right]\right)}^{2}}{\sum_{n=0}^{N-1}x^{2}\left[n\right]}}$$

where *N* is the length of the simulated signals, *x* is the original signals, *y* is the observed signals and *x̂* is the estimation of the original signals through the calculation of NL-Means.

**Figure 3.**
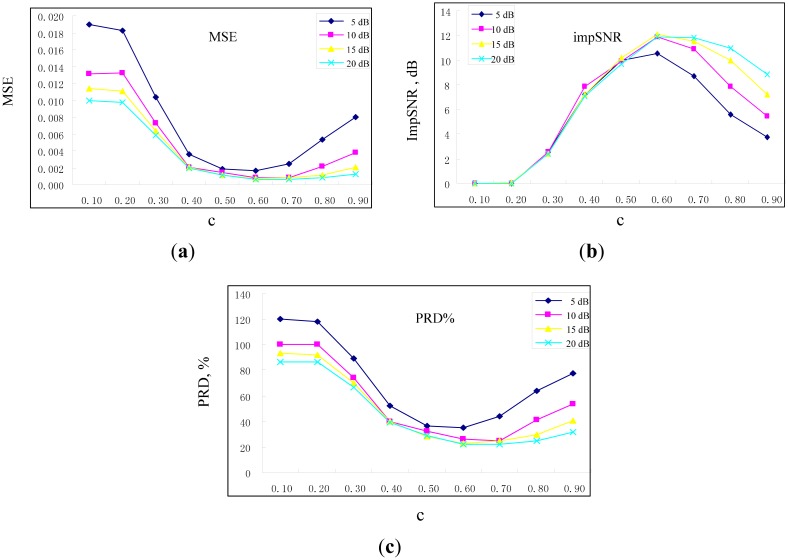
MSE (**a**), impSNR (**b**) and PRD% (percent distortion) (**c**) *versus c*, when separately adding 5 dB, 10 dB, 15 dB and 20 dB white Gaussian noise.

Figure 3 shows different evaluation indexes when fast NL-Means is adapted to the different noises. Here, the *c* is gradually increased from 0.10 to 0.90, and the noise decibel is increased from 5 dB to 20 dB at the same time. From Figure 3, the value of *c* has an optimal solution. In the three evaluation indexes, when λ = 0.6 σ, a lower MSE and PRD% can be obtained; meanwhile, *impSNR* obtains the maximum value. Therefore, the parameters of fast NL-Means for these simulated fault signals of rolling bearings are determined as *P* = 100, *K* = 4000 and λ = 0.6 σ.

**Figure 4.**
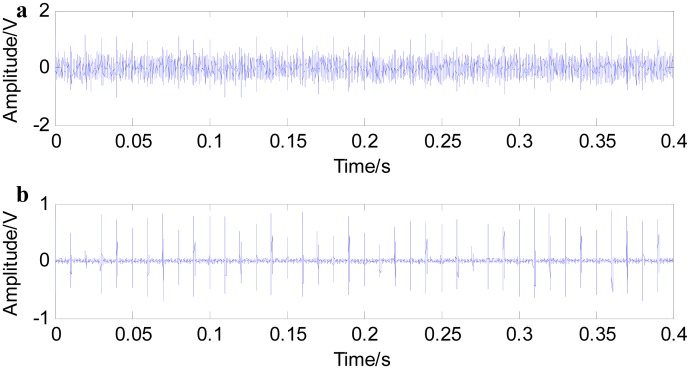
Simulated fault signal of rolling bearing with *SNR* = 2 dB (**a**); and filtered signal with fast NL-Means (**b**).

**Figure 5.**
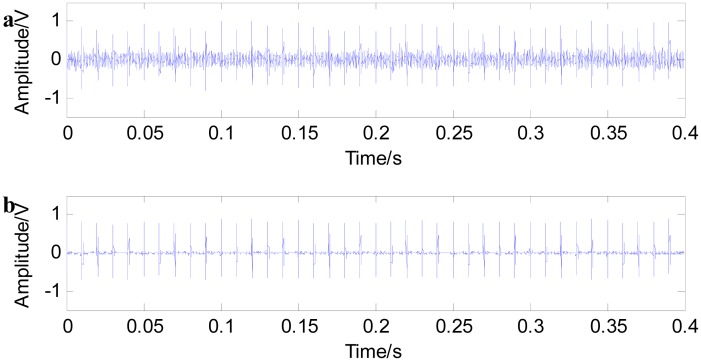
Simulated fault signal of rolling bearing with *SNR* = 20 dB (**a**); and filtered signal with fast NL-Means (**b**).

Figure 4 shows the noise reduction effect of fast NL-Means. The pulse signals are submerged in noises, and the signal peaks are not obvious when adding a signal-to-noise ratio of 2 dB (A) Gaussian white noise (in this paper, it is assumed that there is only one noise type, Gaussian white noise). It is difficult to judge fault impact characteristic information only from the time domain waveform. Figure 4a,b shows the filtered signals with fast NL-Means. From Figure 4, it is noted that with the help of fast NL-Means, the fault characteristic peaks embody the noise signals. Figure 5 shows that a better result can be obtained when reducing the strength of the noises.

**Figure 6.**
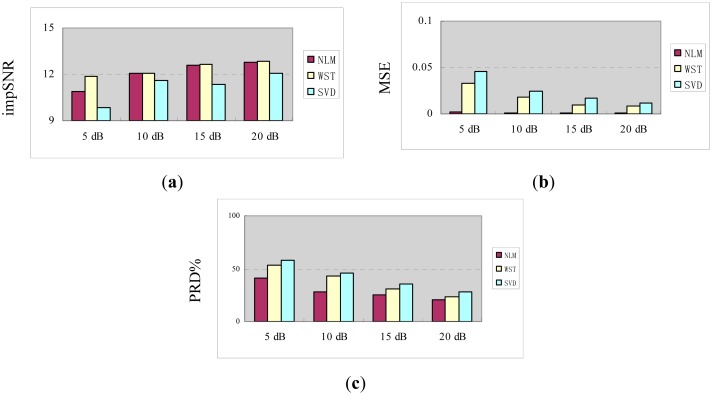
impSNR (**a**), MSE (**b**) and PRD% (**c**) of the simulated fault signals. WST, wavelet soft thresholding; SVD, wavelet soft thresholding method.

In order to further the instructions, Figure 6 shows all three evaluation indexes calculated across the simulated fault signals of the rolling bearing as a function of SNR. The figure also shows the results for the other two algorithms: one is a wavelet soft thresholding method (WST), while the other is a singular value decomposition denoising method (SVD). From Figure 6, NL-Means achieves lower MSE and PRD% than the other methods, when the SNRs growth NL-Means get closer to the original signal.

**Figure 7.**
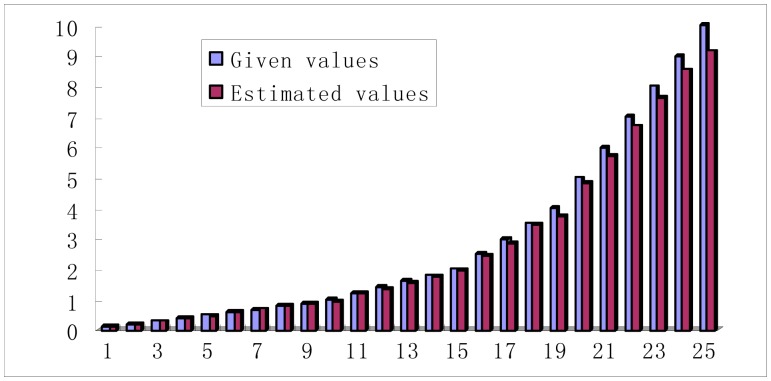
The noise variance estimation *versus* the given values.

For the simulation signals, the noise variance is given, which can be estimated in practice. Many scholars have conducted a lot of research on noise estimation. However, most of the research is based on the wavelet domain. A simple noise variance estimation method in this paper is put forward to reduce the computing time. Gaussian noise is subject to normal distribution *N*(0, σ), and it can be seen as combined random variables, which are made up by a series of small random variable in some cases. From Figure 2b, it can be seen that there are many flat areas on bearing signals, and the variances can be directly regarded as noise variances for those flat areas. Therefore, bearing signals can be cut into many small pieces with the same size, and all of the variance of the small pieces is calculated and the smallest values averaged (the number should not be less than three and more than 1% of the total). For the simulated rolling bearing signals, this method is used to for noise estimation. The experimental results are given in Figure 7. This method caused a very small percentage error, and the estimated values are almost equal to the given values.

## 4. Rolling Bearing Fault Diagnosis Based on Fast NL-Means and Envelop Spectrum

In the rolling bearing fault signal analysis, the fault location rotates with the rolling bearings, so fault signals present a modulation phenomenon. In order to facilitate extracting fault characteristic information, the signal with envelope demodulation analysis need to be filtered. At present, a commonly-used demodulating method is the Hilbert transform; it can demodulate the low frequency target signal from the high carrier frequency. Through envelop spectrum analysis with Hilbert transform, rolling bearing fault features can be accurately extracted.

### 4.1. Envelop Spectrum Analyses with Hilbert Transform

Given a time series, *x*(*t*) meets the typical modulation patterns. The algorithm of envelop analysis with Hilbert transform can be expressed as follows:

(12) $$x\left(t\right)=A\left(t\right)\times \cos\left(2\pi f_{h}t+\varphi \right)$$

where *A*(*t*) is the low frequency modulation signal, cos(2π*f_h_t* + *φ*) is a high frequency carrier signal, *f_h_* is the carrier frequency and φ is the phase modulation information.

To get the Hilbert transform *x̃*(*t*):

(13) $$\tilde{x}\left(t\right)=H\left[x\left(t\right)\right]=f\left(t\right)*\frac{1}{\pi t}=\frac{1}{\pi }\int_{-\infty }^{\infty }\frac{f\left(\tau \right)}{t-\tau }d\tau$$

In Equation (13)*H* is the Hilbert transform and * is convolution. The Hilbert transform is equal to the process of the filter, and the unit impulse response of the filter is 1/π. The time series *x*(*t*) and its Hilbert transform *x̃*(*t*) have orthogonality. Therefore, for the time series *x*(*t*), its orthogonal component *x̃*(*t*) = *A*(*t*) sin(2π*f_h_t* + φ) is introduced to construct a complex analytic signal *g*(*t*):

(14) $$g\left(t\right)=x\left(t\right)+j\tilde{x}\left(t\right)\equiv A\left(t\right)e^{i\varphi }e^{j2\pi f_{h}t}$$

The envelop signal *A*(*t*) of the times series *x*(*t*) can be calculated by acquiring the absolute value of the analytical signal. Eventually, the envelope signal can be expressed as:

(15) $$A\left(t\right)=\sqrt{x^{2}\left(t\right)+{\tilde{x}}^{2}\left(t\right)}$$

Therefore, applying spectrum analysis to envelop signal *A*(*t*), the fault signal features can be obtained accurately.

### 4.2. The Proposed Method for Rolling Bearing Diagnosis

**Figure 8.**
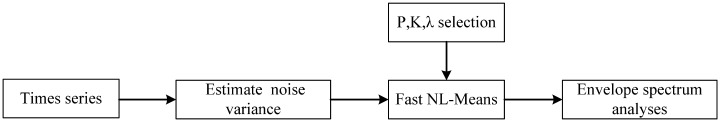
The scheme diagram of fast NL-Means and envelop spectrum.

When faults occur on a rolling bearing, some of the characteristic frequencies will clearly appear in the envelop spectrum. These characteristic frequencies can be used for rolling bearing fault diagnosis, but they are not obvious under the interference of noise. The NL-Means denoising results are not the presentation of mathematical functions, but the good reduction of original signals, so this method consequently causes a small amount of distortion. In sum, combined with fast NL-Means filtering and envelope spectrum analysis, the proposed approach in this paper can be expressed as follows, and the scheme diagram is shown in Figure 8:

(1) Given a rolling bearing signal, cut it into small pieces to estimate the noise variance.
(2) Observe the signals and determine the parameters of fast NL-Means.
(3) Apply fast NL-Means to the filter in order to get the filtered signals from the noises.
(4) Conduct the Hilbert transform with denoising signals to get the envelope wave.
(5) Practice envelope spectrum analysis on the envelope wave to get the characteristic frequencies.

## 5. Experiment

From Figures 4 and 5, it can be seen that the fast NL-Means is very effective. Fast NL-Means can successfully restore the original signals. Here, we adopt the data from the Case Western Reserve University Bearing Data Center website, which provides access to ball bearing test data of normal and faulty bearings. Motor bearings are seeded with faults using electro-discharge machining (EDM). We use the #211 inner race data and the #236 outer ring data that were collected for 12,000 samples/s and at 1730 rpm for drive-end bearing experiments. The type of tested rolling element bearing is 6205-2RS JEM SKF. Additionally, its corresponding parameters are displayed in Table 2. According to the bearing characteristic frequencies in Table 3, the bearing characteristic frequencies are calculated and displayed in Table 4.

**Figure 9.**
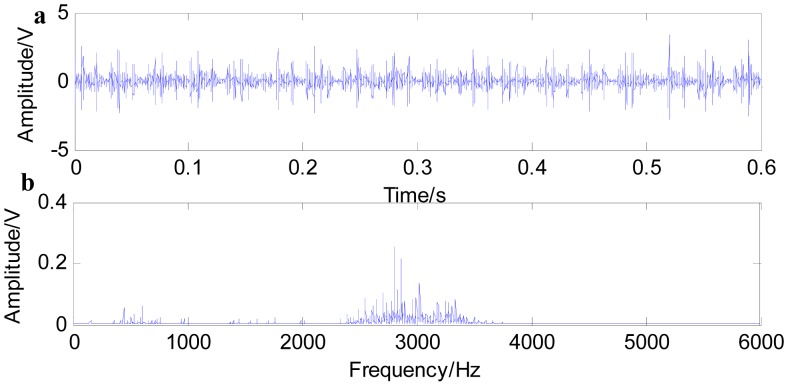
Time waveform of the original signal with an inner race defect (**a**); and the spectrum of the original signal (**b**).

**Figure 10.**
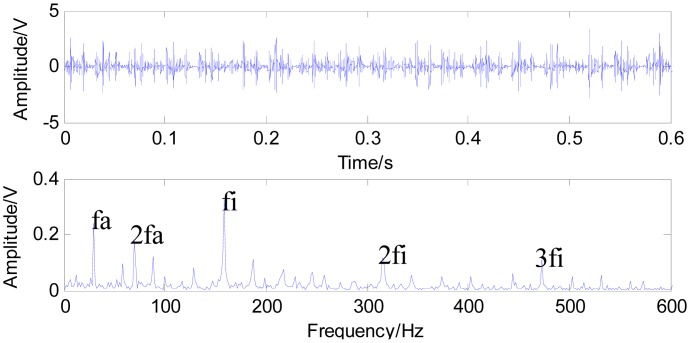
Time waveform of filtered signal with an inner race defect using fast NL-Means (**a**); and the envelope spectrum of the filtered signal (**b**).

In this paper, we conduct fast NL-Means and the envelop spectrum process on the rolling bearing signals with the inner race defect and outer race defect separately. Figure 9 shows the time waveform of the original signals with an inner race defect (a) and the spectrum of the original signals (b). The characteristics of impact are not obvious under the condition that the bearing characteristic information is submerged by the noises in Figure 9a. The energy mainly concentrates in the high frequency of the resonance wave in the spectrum. Firstly, the noise variance is estimated, then fast-NL-Means is conducted with the original signals. Figure 10a shows the time waveform of filtered signals. We note that the filtered signals have obvious impact characteristics. Fast NL-Means retains the high amplitude area and reduces the noise interferences better. The analysis of the envelope shows some obvious characteristic frequencies of this rolling bearing. In Figure 10b, the rotating speed frequency *f_a_* = 29 Hz, and the inner ring failure frequency *f_i_* = 158 Hz are clear, and we also find the frequency multiplications 2*f_i_* and 3*f_i_*. At the same time, the failure frequency amplitude is obvious; thus, we can diagnose that a bearing inner ring fault happened.

**Figure 11.**
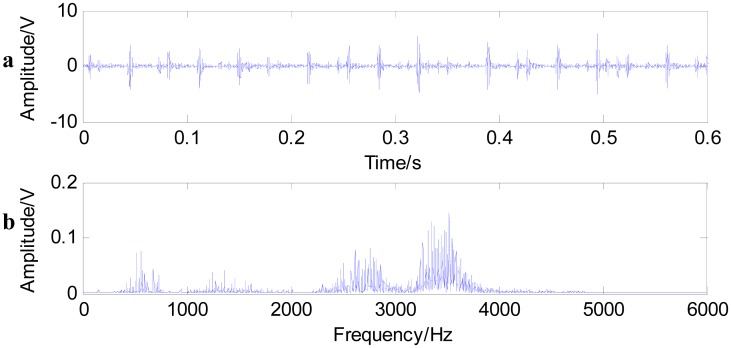
Time waveform of the original signal with an outer race defect (**a**); and the spectrum of the original signal (**b**).

**Figure 12.**
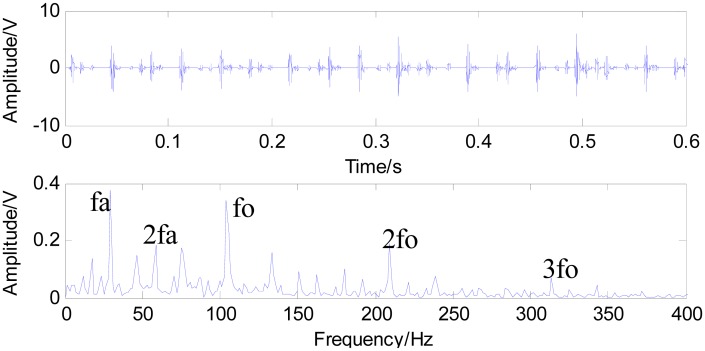
Time waveform of the filtered signal with an outer race defect using fast NL-Means (**a**); and the envelope spectrum of the filtered signal (**b**).

Figure 11 shows the time waveform of the original signals with an outer race defect (a) and the spectrum of the original signals (b). From Figure 11a, it can be seen that the time domain waveform is desultory and that a burr is prominent. However, the signal energy concentrates on the high order vibration mode, and low frequency characteristics of signals are drowned out by the noises in Figure 11b. Figure 12 shows the time waveform of filtered signals with an outer race defect by using fast NL-Means (a) and the envelope spectrum of the filtered signals (b). From Figure 12a, we can see that high amplitude areas are better preserved, and the noise reduction is obvious. Through applying the proposed approach to the original signals, the outer race defect can be found, then envelop spectrum analysis is performed. From Figure 12b, the rotating speed frequency *f_a_* = 29 Hz and the outer ring failure frequency *f_o_* = 105 Hz, the frequency multiplication 2 *f_o_* and 3 *f_o_* are clear. Meanwhile, the failure frequency amplitudes are obvious. Thus, we can diagnose that a bearing outer ring fault happened.

**Figure 13.**
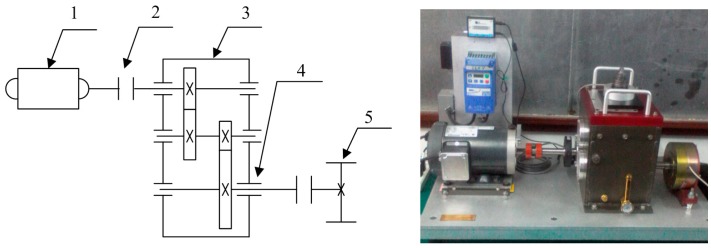
Experimental apparatus. 1, variable speed drive; 2, torque transducer and encoder; 3, parallel shaft gearbox; 4, test point; 5, programmable magnetic brake.

In order to further verify the effectiveness of the proposed method in this paper, we conduct the experiment on the Drivetrain Diagnostics Simulator, which is provided by SQi in the USA. The experimental device is shown in Figure 13. The drivetrain consists of a 2-stage planetary gearbox, a 2-stage parallel shaft gearbox with rolling or sleeve bearings, a bearing loader and a programmable magnetic brake. Here, normal gear and a defect bearing with an outer ring pitting fault are used for testing. The sampling frequency is 2000 Hz, and the rotating speed frequency is 57 Hz. Because of the characteristics of the three-phase asynchronous motor, the actual rotating speed frequency fluctuates slightly. In the test, we measured the rotating speed frequency of the input shaft *f_1_* = 57 Hz through the tachometer and the actual rotating speed frequency of the intermediate shaft *f_2_* = 15.6 Hz and the output shaft *f_3_* = 6.2 Hz. The defect bearing is the FAFNIR deep groove ball bearing ER-16k. According to the bearing parameters and the gearbox transmission rule, the characteristic frequency of ER-16k with an outer ring fault *f_0_* = 3.572, *f_1_* = 188.2 Hz.

**Figure 14.**
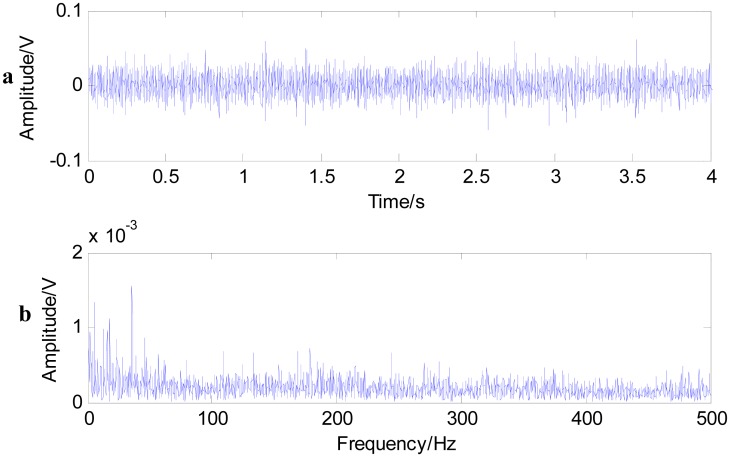
The time waveform of the original signal with an outer race defect (**a**); and the envelop spectrum of the original signal (**b**).

**Figure 15.**
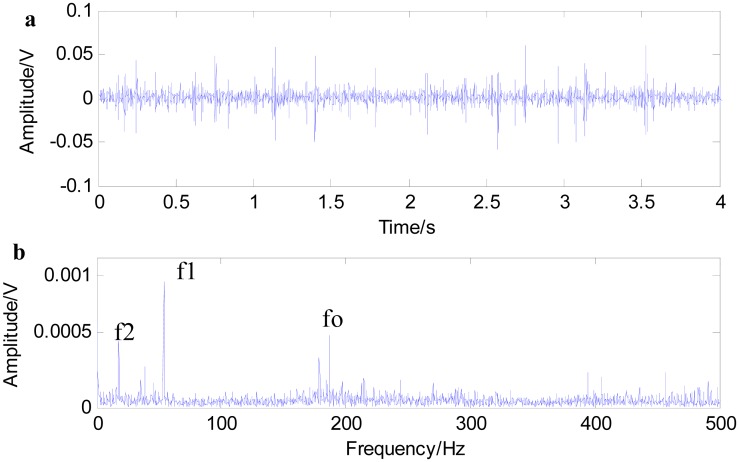
Time waveform of the filtered signal with an outer race defect using fast NL-Means (**a**); and the envelope spectrum of the filtered signal (**b**).

Figure 14 shows the time waveform of the original signals with an outer race defect (a) and the envelop spectrum of the original signals (b). Due to the noise interferences, the original signal waveform is desultory, and the impact characteristic peaks are not obvious. The characteristic frequencies cannot be found in the envelope spectrum. Figure 15 shows the time waveform of filtered signals with an outer race defect by using fast NL-Means (a) and the envelope spectrum of the filtered signals (b). The high amplitude areas are better preserved, and the impact characteristic peaks are obvious, because the noises are suppressed to a certain extent. On the envelope spectrum, we find the rotating speed frequency of input shaft *f_1_* and intermediate shaft *f_2_*. We can also find the outer ring failure characteristic frequency *f_o_*. The characteristic frequency amplitudes are obvious and have no interference frequency components. For this reason, we can diagnose that the bearing outer ring fault happened.

## 6. Conclusions

Nowadays, the fault diagnosis of rolling bearing signals in a strong, noisy, jamming environment has received considerable attention. A novel method based on the fast NL-Means algorithm and envelops spectrum analysis dedicated to this difficult task is proposed in this paper. The patch-based method (NL-Means algorithm) uses the weighting average of the similar patches for denoising. By optimizing the parameters, the noise component can be optimally eliminated. Then, envelop spectrum analysis is applied on the denoising signals. Even more exciting, the characteristic frequencies can be clearly obtained. The experimental results reveal that the proposed method performs excellently at denoising for the fault diagnosis of rolling bearing. What is more, a noteworthy aspect is that the high amplitude areas of the bearing signals obtain a better retention. However, there are some legacy problems to be solved; for example, NL-Means is short of the capacity to distinguish similar patches, which have different fine structures, and the lack of patches would cause an insufficient average. These limitations would provide promising avenues for our future work.

## Figures and Tables

**Table 1.** The process of fast NL-Means.

**Given a Time Series of Vibration Signals** *y* = [*a*_1_,*a*_2_,*a*_3_,…*a_N_*]. ***N* Denotes the Length of the Time Series.**
(1) To determine the parameters, half of the search domain size *K*, half of patch size *p*, bandwidth parameter λ.
(2) Do summation over 2*K* + 1; calculate all of the values of *S_dx_* using Equation (4). Then, increase *d_x_* gradually by each step.
(3) Calculate all of the values of distance (point-by-point MSE) *d^2^* using Equations (5) and (6). Calculate all of the values of weights ω using Equation (3).
(4) Finally, apply Equation (2) to realize the process of the filter.

**Table 2.** Rolling element bearing parameters.

**Rolling Element Bearing Parameters of 6205-2RS JEM SKF**					
**Inside Diameter** *d*_1_ **(mm)**	**Outside Diameter** *d*_2_ **(mm)**	**Ball Number *n***	**Ball Diameter** *d_r_* **(mm)**	**Contact Angle** *α*	**Pitch Diameter** *D_w_* **(mm)**
25	52	9	7.9	0	46.4

**Table 3.** Bearing characteristic frequencies.

**Fault Location**	**Failure Frequency**
Defect on inner race (BPI)	$f_{i}=\frac{n}{2}\left(1+\frac{d_{r}}{D_{w}}\cos\alpha \right)f_{a}$
Defect on outer race (BPO)	$f_{o}=\frac{n}{2}\left(1+\frac{d_{r}}{D_{w}}\cos\alpha \right)f_{a}$
Defect on cage (FT)	$f_{t}=\frac{1}{2}\left(1-\frac{d_{r}}{D_{w}}\cos\alpha \right)f_{a}$
Defect on ball (BS)	$f_{b}=\frac{d_{r}}{2D_{w}}\left[1-{\left(\frac{d_{r}}{D_{w}}\right)}^{2}\cos^{2}\alpha \right]f_{a}$

(*f_a_* is the rotation frequency of the rolling bearing. Here, *f_a_* = 29.17 Hz).

**Table 4.** Characteristic frequencies of rolling element bearing 6205-2RS JEM SKF (Hz).

**Characteristic Frequencies (Hz)**				
*f_a_*	*f_o_*	*f_i_*	*f_b_*	*f_t_*
29.17	104.56	157.94	137.48	11.62
